# *Rhizobium leguminosarum *bv. *trifolii rosR *is required for interaction with clover, biofilm formation and adaptation to the environment

**DOI:** 10.1186/1471-2180-10-284

**Published:** 2010-11-11

**Authors:** Monika Janczarek, Jolanta Kutkowska, Tomasz Piersiak, Anna Skorupska

**Affiliations:** 1 Department of Genetics and Microbiology, University of M. Curie-Skłodowska, Akademicka 19, 20-033 Lublin, Poland; 2 Department of Comparative Anatomy and Anthropology, University of M. Curie-Skłodowska, Akademicka 19, 20-033 Lublin, Poland

## Abstract

**Background:**

*Rhizobium leguminosarum *bv. *trifolii *is a symbiotic nitrogen-fixing bacterium that elicits nodules on roots of host plants *Trifolium *spp. Bacterial surface polysaccharides are crucial for establishment of a successful symbiosis with legumes that form indeterminate-type nodules, such as *Trifolium*, *Pisum*, *Vicia*, and *Medicago *spp. and aid the bacterium in withstanding osmotic and other environmental stresses. Recently, the *R. leguminosarum *bv. *trifolii *RosR regulatory protein which controls exopolysaccharide production has been identified and characterized.

**Results:**

In this work, we extend our earlier studies to the characterization of *rosR *mutants which exhibit pleiotropic phenotypes. The mutants produce three times less exopolysaccharide than the wild type, and the low-molecular-weight fraction in that polymer is greatly reduced. Mutation in *rosR *also results in quantitative alterations in the polysaccharide constituent of lipopolysaccharide. The *rosR *mutants are more sensitive to surface-active detergents, antibiotics of the beta-lactam group and some osmolytes, indicating changes in the bacterial membranes. In addition, the *rosR *mutants exhibit significant decrease in motility and form a biofilm on plastic surfaces, which differs significantly in depth, architecture, and bacterial viability from that of the wild type. The most striking effect of *rosR *mutation is the considerably decreased attachment and colonization of root hairs, indicating that the mutation affects the first stage of the invasion process. Infection threads initiate at a drastically reduced rate and frequently abort before they reach the base of root hairs. Although these mutants form nodules on clover, they are unable to fix nitrogen and are outcompeted by the wild type in mixed inoculations, demonstrating that functional *rosR *is important for competitive nodulation.

**Conclusions:**

This report demonstrates the significant role RosR regulatory protein plays in bacterial stress adaptation and in the symbiotic relationship between clover and *R. leguminosarum *bv. *trifolii *24.2.

## Background

Nitrogen-fixing symbiotic bacteria, commonly known as rhizobia, employ a variety of strategies which allow them to exist in the soil and adapt to various environmental conditions prior to infecting leguminous plant hosts. Rhizobial cell surface components, exopolysaccharide (EPS) and lipopolysaccharide (LPS), play an important role in determining the symbiotic competence of rhizobia, root tissue invasion and induction of nitrogen-fixing nodules on host plants forming indeterminate-type nodules, such as *Pisum*, *Trifolium*, *Vicia*, and *Medicago *spp. [1-4]. Acidic EPSs secreted in large amounts by rhizobia are species-specific compounds consisting of common sugars substituted with non-carbohydrate residues [1,4-6]. EPS of *Rhizobium leguminosarum *is a heteropolymer consisting of octasaccharide subunits composed of five glucose residues, one galactose, and two glucuronic acid residues, additionally decorated with acetyl, pyruvyl, and 3-hydroxybutyryl groups [7,8]. EPS-deficient mutants or those with an altered LPS structure are impaired in nodule cell invasion and nitrogen fixation [1,6,9-11]. Biosynthesis of EPS in *R. leguminosarum *is a multi-step process requiring the expression of several *pss *genes, located in the major EPS cluster on the chromosome [12,13]. This region includes *pss *genes encoding specific glycosyl transferases, epimerases and deacetylases involved in the biosynthesis of EPS repeating units, genes encoding proteins engaged in the polymerization and transport of EPS, and other genes that code for EPS modifying enzymes [12,13]. As has been established for *R. leguminosarum *and *Sinorhizobium (Ensifer) meliloti*, EPS plays an important role in biofilm development, being the major matrix component [14-17]. A mutation in *R. leguminosarum pssA *encoding the first IP-glucosyl transferase essential for EPS synthesis completely abolishes biofilm development [14,18]. Glycanases PlyA and PlyB secreted via the PrsD-PrsE type I secretion system are responsible for EPS modification and biofilm formation. PlyA and PlyB cleave mature EPS. Exopolysaccharides produced by *prsD*, *plyB*, and *plyBplyA *mutants form significantly longer polymers than the wild type [19,20]. Besides glycanases, RapC, RapA1, and RapA2 agglutinins engaged in the adhesion and aggregation of rhizobia are secreted via the PrsD-PrsE type I secretion system [14,21,22].

In a previous study, a *rosR *gene encoding a positive transcriptional regulator of EPS synthesis was identified in *R. leguminosarum *bv. *trifolii *[23]. The chromosomally located *rosR *shares significant identity with *rosR *of *Rhizobium etli *[24], *mucR *of *Sinorhizobium meliloti *[25], *ros *of *Agrobacterium tumefaciens *[26], and *rosAR *of *Agrobacterium radiobacter *[27]. Transcriptional regulators encoded by these genes belong to the family of Ros/MucR proteins which possess a Cys_2_His_2 _type zinc-finger motif and are involved in positive or negative regulation of EPS synthesis. A genome-wide genetic screening has revealed that *R. etli rosR *affects the expression of about fifty genes, among them those responsible for the synthesis, polymerization, and transport of surface polysaccharides [28]. *rosR *of *R. leguminosarum *bv. *trifolii *encodes a protein of 143 aa (15.7 kDa) containing a zinc-finger motif in its C-terminal domain that binds a 22-bp-long consensus sequence called the RosR-box, which is located in the *rosR *upstream region. Besides the RosR-box, several regulatory sites have been identified in the *rosR *upstream region, including two P1 and P2 promoters and three motifs resembling the *E. coli *cAMP-CRP binding site, indicating a complex regulation of *rosR *expression [23,29]. RosR binding to the RosR-box negatively regulates transcription of its own gene [23]. In the presence of glucose, the transcriptional activity of the *rosR *is significantly reduced, showing that the expression of this gene is regulated by catabolic repression.

*rosR *mutation in *R. leguminosarum *bv. *trifolii *causes a substantially diminished EPS production and ineffective symbiosis with clover [30]. In contrast, although an *R. etli rosR *mutant also formed colonies with altered morphology, it retained the ability to elicit nitrogen-fixing nodules on *Phaseolus vulgaris*, which forms determinate-type nodules [24]. The nodulation competitiveness of this mutant, however, was greatly reduced and, for this reason, *rosR *was assumed to be a determinant of *R. etli *competitiveness.

In this study, we describe pleiotropic phenotypes of *rosR *mutants, which are characterized by an increased sensitivity to osmotic stresses, detergents, and antibiotics that affect peptidoglycan synthesis. These mutants produce significantly less EPS than the wild type and form an altered biofilm on polystyrene surfaces. Moreover, the mutation in *rosR *affects symbiotic performance, strikingly decreasing bacterial attachment to clover root hairs and formation of infection threads.

## Results

### *R. leguminosarum *bv. *trifolii rosR *mutants

**Figure 1:**
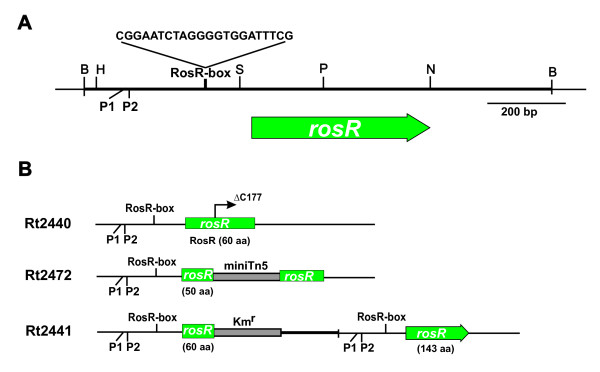
**Physical map of *R. leguminosarum* bv. trifolii *rosR* gene and genomic organization of *rosR* mutants. **Physical and genetic map of pB31 plasmid carrying the *rosR *gene of *Rhizobium leguminosarum *bv. *trifolii *24.2 (A). (B) The genomic organisation of the Rt2440, Rt2441, and Rt2472 mutants. The heavy line indicates the vector part in the Rt2441 integration mutant. B- *Bam*HI, H- *Hin*dIII, S- *Sal*I, P- *Pst*I, N- *Not*I. P1 and P2 are promoter sequences of the rosR gene, and the RosR-box sequence is the target site recognized and bound by RosR protein.

Recently, we described *R. leguminosarum *bv. *trifolii *24.2 derivatives mutated in the *rosR *open reading frame (Rt2440 and Rt2472) [23,29]. In this study, using integrative mutagenesis, the Rt2441 mutant was constructed in which a fragment containing the 5'-end regulatory region and the first 60 nucleotide triplets for RosR was integrated 360 bp upstream of genomic *rosR *ORF, just before the P1 promoter (Figure 1B). We wanted to examine the effect of duplication of regulatory sequences consisting of two RosR-boxes, which constitute the sites of interaction with the zinc finger motif of the RosR transcription factor, on several phenotypic and symbiotic properties of the mutant.

The two previously described *rosR *mutants (Rt2440 and Rt2472) were also evaluated in some assays (Figure 1). The Rt2440 mutant has 1 bp deletion (ΔC177) in *rosR *ORF, resulting in a frameshift mutation and a subsequent synthesis of RosR with a non-native amino acid sequence downstream of the mutation [23]. The Rt2472 mutant was obtained by gene replacement mutagenesis using the mini-Tn*5 *transposon inserted between 151-152 nt of *rosR *ORF [30].

### *R. leguminosarum rosR *mutants are defective in symbiotic efficiency and competitiveness

All *rosR *mutants demonstrated similar colony phenotypes; they formed characteristic dry, wrinkled colonies with many clumps on 79CA agar medium (data not shown). Clover inoculated with the *rosR *mutants formed nodules with a 7-day delay, and their number was about two-fold lower in comparison to the wild type (Table 1). Inoculated plants turned yellowish, which indicated inefficient symbiosis, and the fresh mass of shoots was, on average, 69.2% of the aerial parts of plants inoculated with Rt24.2. Irrespective of the type of *rosR *mutation, wild type copies of *rosR *carried on the low-copy plasmid pRC24 fully complemented the mutation. Transconjugants were mucoid (EPS^+^), and clover inoculated with the clones demonstrated symbiotic phenotypes similar to the wild type (Table 1).

**Table 1:** *rosR *mutation affects symbiotic properties and EPS production of *R. leguminosarum *bv. *trifolii *24.2. Defects are fully complemented by the wild-type *rosR *copy.

Strain/plasmid	**Nodule no. per plant**^**a**^	**Shoot weight (mg/plant)**^**a **^		**EPS (mg/mg)**^**b**^
		(fresh wt)	(dry wt)	
Rt2440	5.1 ± 1.9	42.4 ± 11.4	4.3 ± 0.15	0.31 ± 0.03
Rt2441	6.2 ± 2.1	44.8 ± 10.2	4.9 ± 0.20	0.36 ± 0.04
Rt2472	4.9 ± 1.7	43.2 ± 7.7	4.2 ± 0.10	0.30 ± 0.03

Rt2440(pRC24)	12.3 ± 3.1	59.3 ± 12.5	6.1 ± 0.25	1.19 ± 0.07
Rt2441(pRC24)	12.5 ± 3.6	58.8 ± 10.2	6.0 ± 0.2	1.15 ± 0.05
Rt2472(pRC24)	12.7 ± 5.4	61.2 ± 14.2	6.2 ± 0.3	1.21 ± 0.06

Rt24.2 (wild type)	12.8 ± 2.9	62.8 ± 12.1	6.2 ± 0.25	0.97 ± 0.05
Uninoculated clover	-	34.7 ± 6.4	3.8 ± 0.10	-

^a ^Plants were harvested 28 days after inoculation. Given values ( ± standard deviation) are averages of three independent experiments with 20 plants for each treatment.

^b ^- Exopolysaccharide (EPS) production (Glc equivalents in mg/mg of protein).

To study the competitive ability of the Rt2472 and the Rt2441 mutants, clover seedlings were inoculated with mixtures of each *rosR *mutant with Rt24.2 wild type in various proportions. For both mutants, in the case of a 1:1 strain ratio, the nodules were colonized exclusively by the Rt24.2 wild type. In 10:1, 100:1, and 1000:1 strain mixtures, the percentage of nodules occupied by the Rt2472 mutant was 1%, 2.5% and 9% of the sampled nodules, respectively (details not shown). The Rt2441 mutant demonstrated a similar decrease in competitiveness: the percentages of occupied nodules were 1%, 4.4%, and 11.1% in the 10:1, 100:1, and 1000:1 mixtures, respectively. The results indicated that *rosR *mutation substantially reduced the nodulation competitiveness of *R. leguminosarum *bv. *trifolii *24.2.

### *rosR *mutants are altered in surface polysaccharides

Non-mucoid colonies formed by the *rosR *mutants indicated that the strains produced reduced amounts of surface polysaccharides. The amounts of EPS secreted by Rt2440, Rt2441 and Rt2472 were estimated to be about 30% of the amount formed by the wild type (Table 1). Rt2441, bearing a truncated *rosR *and an additional wild type copy of the gene, demonstrated the negative dominant nature of *rosR *mutation.

**Figure 2:**
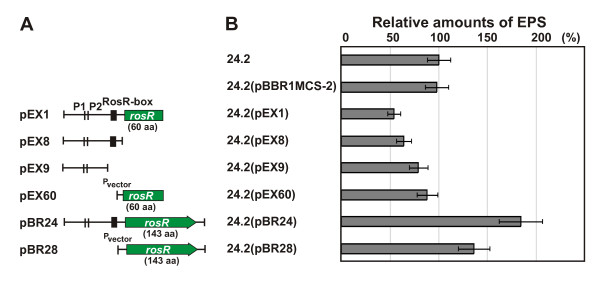
**The effect of additional copies of different regulatory *rosR *sequences on the EPS production by *R. leguminosarum***. Data shown are the means of three replicates ± SD.

To test the negative dominant effect on EPS production observed in Rt2441, plasmids containing different fragments of the regulatory region and *rosR *were constructed on pBBR1MCS backbone and introduced into Rt24.2 (Figure 2). The pEX1 plasmid containing the same fragment as in the Rt2441 mutant genome exerted a strong negative effect on EPS production, decreasing EPS synthesis to 54% of the control (Figure 2). Rt24.2(pEX8), containing exclusively the *rosR *upstream region with the RosR-box, produced 64% EPS of the wild type strain, but Rt24.2(pEX60), which contained only the shortened *rosR *ORF, produced 88% of Rt24.2 EPS. Only after introducing full-length copies of *rosR *into Rt24.2 (especially under its own promoter, on plasmid pBR24), the negative dominant effect had been overcome, with the increase of EPS synthesis up to 183% of the control. These results suggested that additional copies of the *rosR *upstream region with the RosR-box sequence, rather than RosR protein deprived of the C-terminal DNA binding domain, affected the level of EPS production. Most likely, the positive regulation of EPS synthesis by RosR depends on an equilibrium between *rosR *regulatory sequences and the amount of RosR. These results explain, to some extent, the phenotype of the Rt2441 mutant.

**Figure 3:**
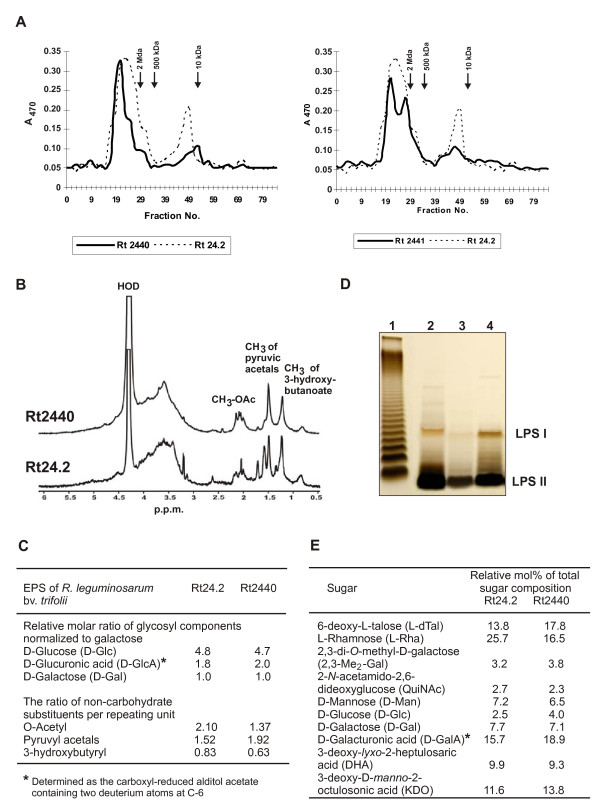
**Gel filtration chromatography of exopolysaccharides (EPS) produced by the *R. leguminosarum *bv. *trifolii *24.2 wild type and the *rosR *mutants (Rt2440 and Rt2441)**. (A) EPS was fractionated on a Bio-Gel A-5m column, as described in the Methods. The retention times of molecular mass markers: dextran blue (2 MDa), dextran T250 (250 kDa), and dextran T10 (10 kDa) are indicated by arrows. (B) A 500 MHz ^1^H-NMR spectrometry analysis of the *R. leguminosarum *wild type and the *rosR *mutant (Rt2440). (C) The glycosyl components and non-carbohydrate substituents of EPS from the wild type and the mutant Rt2440. (D) Silver-stained Tricine SDS-PAGE profiles of LPS from the wild type and the *rosR *mutants. LPSs (2 μg) were loaded in 2 μl sample buffer. Lanes: 1- *Salmonella enterica *sv. Typhimurium (Sigma), 2- wild type Rt24.2, 3- Rt2440, 4- Rt2441. LPS I, high-molecular-weight LPS; LPS II, low-molecular-weight LPS. (E) The glycosyl composition of polysaccharides lacking lipid A released from LPS by mild acid hydrolysis from the wild type and the *rosR *mutant (Rt2440).

EPSs isolated from the Rt24.2 wild type and Rt2440 and Rt2441 *rosR *mutants were fractionated by gel permeation chromatography on a Bio-Gel A-5m column, and two fractions of EPS with significantly different molecular weights were obtained (Figure 3A). The ratio of high-molecular-weight (HMW) to low-molecular-weight (LMW) fractions was 68%:32% in the EPS of Rt24.2 wild type. In the Rt2440 and Rt2441 *rosR *mutants, a considerable change was observed in the HMW to LMW EPS ratio in favor of HMW, i.e., 79%:21% and 76%:24%, respectively. To establish the sugar composition of EPS of the wild type and the *rosR *mutant, peak samples from Bio-Gel A-5m chromatography (Figure 3A) were evaluated for monosaccharide composition by GC-MS. The glucose/glucuronic acid/galactose ratio was found to be approximately 5:2:1, which is characteristic of the acidic EPS of *R. leguminosarum *(Figure 3C). Additionally, non-carbohydrate substituents in the EPS of Rt2440 and Rt24.2 wild type were determined (Figure 3B-C). EPS secreted by the *rosR *mutant had a lower level of O-acetyl and 3-hydroxybutyryl substitutions and slightly more pyruvyl substitutions in relation to the wild type EPS (Figure 3B).

To investigate whether the *rosR *mutation affected LPS synthesis, LPSs from Rt24.2, Rt2440, and Rt2441 were analyzed by SDS-PAGE (Figure 3D). The LPS of Rt24.2 wild type separated into two intense bands: fast-migrating LPS II representing lipid A and the core oligosaccharide, and slow-migrating LPS I carrying the O antigen [31,32]. The appearance of faintly stained bands in the upper region of the gel indicated the presence of LPS forms with O-chains composed of more polymerized repeating units. LPS of Rt2440 had a similar profile; however, the intensity of the individual bands was much weaker than for Rt24.2 (Figure 3D). High-molecular-weight LPS (LPS I) from the *rosR *mutant migrated slightly faster than LPS I of the wild type. In order to assign these changes, the glycosyl compositions of polysaccharides (PSs) obtained from the wild type and the Rt2440 mutant LPSs by mild acid hydrolysis were examined (Figure 3E). It was established that the sugar composition of both PSs was the same, although some differences in the amounts of individual components (especially 6-deoxyhexoses) were observed. The ratio of L-rhamnose to 6- L-deoxytalose was 1:1 in PS of the *rosR *mutant as compared to 2:1 in the wild type PS. Our preliminary results (R. Russa, personal communication) indicate that L-rhamnose and 6-L-deoxytalose are compounds of both O-chain repeating units and a non-repeating glycosyl sequence of the outer core region.

### *R. leguminosarum rosR *mutants are more sensitive to some antibiotics, detergents, and osmotic stresses

**Figure 4:**
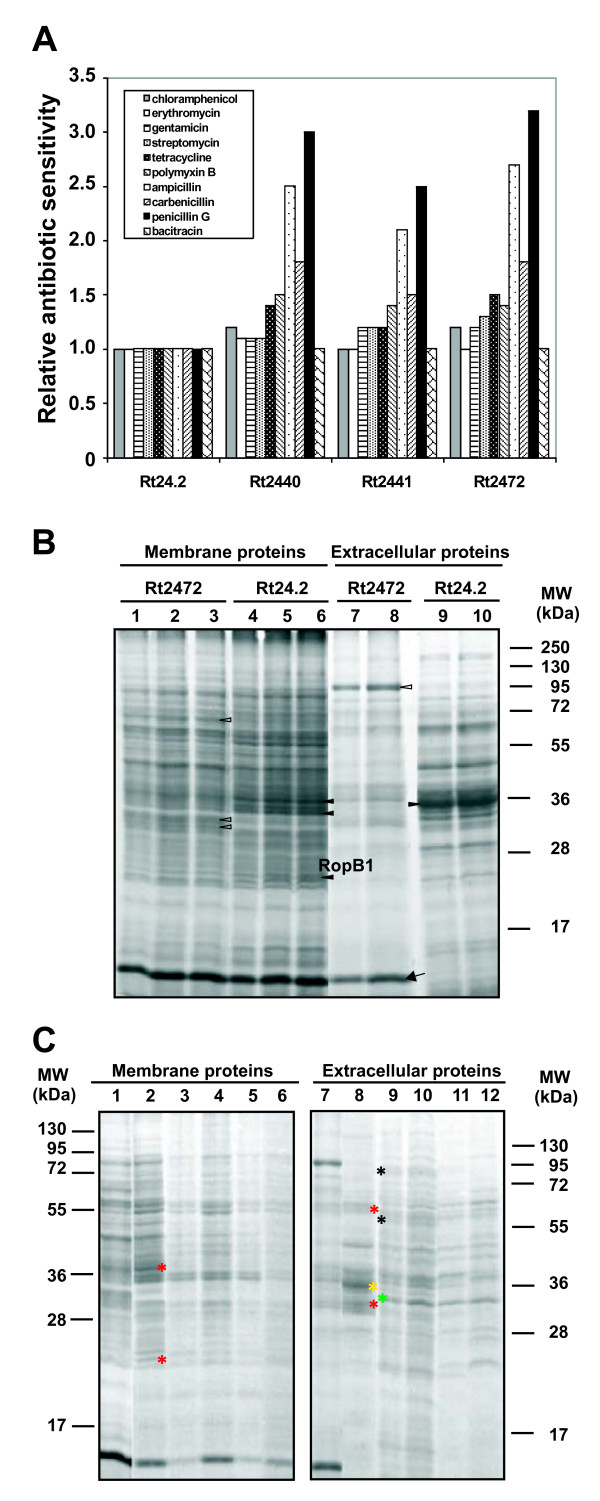
**Sensitivity to antibiotics and profiles of membrane and extracellular proteins of *R. leguminosarum* bv. *trifolii rosR* mutants.** Relative sensitivity of the *R. leguminosarum *bv. *trifolii rosR *mutants to antibiotics, determined by measuring the diameter of growth-inhibition zones (A). The values for the Rt24.2 wild type which were used for result normalization were as follows: chloramphenicol 16.9 ± 1.5 mm, erythromycin 24.0 ± 1.5 mm, gentamicin 22.8 ± 1.8 mm, streptomycin 23.5 ± 2.0 mm, tetracycline 45.2 ± 2.2 mm, polymyxin B 5.5 ± 1.0 mm, ampicillin 9.0 ± 1.0 mm, carbenicillin 24.5 ± 2.5 mm, penicillin G 3.5 ± 0.5 mm, bacitracin 14 ± 2.0 mm. Data shown are means of three replicates. (B) Profiles of membrane and extracellular proteins of the Rt24.2 wild type and Rt2472 *rosR *mutant grown in TY medium. The migration positions of molecular mass markers are shown. Lanes: 1, 2, 3 - Rt2472 membrane protein fraction: 3 μg, 6 μg, and 9 μg, respectively. Lanes: 4, 5, 6 - Rt24.2 wild type membrane protein fraction: 3 μg, 6 μg, and 9 μg, respectively. Lanes: 7, 8 - Rt2472 extracellular protein fraction isolated from 10 ml and 15 ml culture supernatants, respectively. Lanes: 9, 10 - Rt24.2 extracellular protein fraction isolated from 10 ml and 15 culture supernatants, respectively. The symbols indicate prominent proteins which vary apparently in the amount between the *rosR *mutant and the wild type: white triangles - proteins up-regulated in Rt2472 mutant, black triangles - proteins of increased amounts in Rt24.2 wild type, arrow - a protein unique to Rt2472 extracellular protein fraction. (C) Membrane and extracellular protein profiles of the wild type and the *rosR *mutant grown in TY and M1 medium with or without 5 μM exudates. Lane: 1- membrane proteins of Rt2472 grown in TY; 2- membrane proteins of Rt24.2 grown in TY; 3- membrane proteins of Rt24.2 grown in M1; 4 - membrane proteins of Rt24.2 grown in M1 with 5 μM exudates; 5- membrane proteins of Rt2472 grown in M1; 6 - membrane proteins of Rt2472 grown in M1 with 5 μM exudates. In the case of lanes 1 to 6, 5 μg of proteins were used. Lanes 7 and 8 - extracellular proteins isolated from TY supernatant of Rt2472 and Rt24.2 cultures, respectively; Lanes 9 and 10 - Rt24.2 extracellular proteins isolated from M1 and M1 with 5 μM exudates supernatants, respectively; Lanes 11 and 12 - Rt2472 extracellular proteins isolated from M1 and M1 with 5 μM exudates supernatants, respectively. In the case of lines 7 to 12, proteins from 10 ml culture supernatant were used. The asterisks indicate prominent proteins which vary apparently in the amount between TY and M1 media for the wild type and the *rosR *mutant: red asterisks - proteins unique to Rt24.2 and Rt2472 strains growing in TY medium, yellow asterisk - a protein unique to the extracellular protein fraction of Rt24.2 isolated from TY supernatant, green asterisk - a protein uniquely present in extracellular protein fractions of Rt24.2 and Rt2472 isolated from M1 supernatants, black asterisks - proteins present exclusively in the extracellular protein fraction of Rt24.2 isolated from M1 supernatant.

To further characterize the *rosR *mutants, their sensitivity to a wide range of antibiotics, including those responsible for cell wall and protein synthesis inhibition, was examined (Figure 4A). The Rt2440 and Rt2441 mutants demonstrated similar antibiotic sensitivity profiles. The most remarkable difference in their antibiotic sensitivity in relation to the wild type was a 2.5- to 3.4-fold increase in susceptibility to beta-lactams, such as carbenicillin, ampicillin, and penicillin G, which impair peptidoglycan synthesis. Also, a slight increase in the sensitivity to polymyxin B (which perturbs the bacterial cell membrane), tetracycline, and chloramphenicol was detected (Figure 4A). The data suggested some changes in the cell envelope structure of the *rosR *mutants; specifically, the alteration in the LPS and EPS profiles could affect cell wall permeability and, consequently, lead to an increase in susceptibility to several antibiotics [33].

To study the possible cell envelope disturbances linked to the *rosR *mutation, assays of sensitivity to detergents and ethanol were conducted (Table 2). In the presence of SDS, DOC, and ethanol, significant differences in the growth of the *rosR *mutants in relation to the parental strain were observed, except for Rt2441, for which growth was moderately inhibited by ethanol (Table 2).

**Table 2:** Sensitivity of *R. leguminosarum *bv. *trifolii rosR *mutants to detergents, ethanol, and osmotic stress.

Strain	Minimal inhibitory concentration			Hyperosmotic	Hypo-osmotic
			
	SDS (% w/v)	DOC (% w/v)	Ethanol (%v/v)	stress (%)*	stress (%)*
Rt24.2	0.05 ± 0.005	0.10 ± 0.005	4.5 ± 0.28	77.1	51.6
Rt2440	0.02 ± 0.003†	0.030 ± 0.003†	2.3 ± 0.25†	11.5†	13.0†
Rt2441	0.02 ± 0.002†	0.030 ± 0.003†	3.0 ± 0.28	11.9†	15.2†
Rt2472	0.015 ± 0.002†	0.025 ± 0.002†	2.6 ± 0.28†	10.4†	13.3†

* - Strains were grown in TY supplemented with 85 mM NaCl (hyperosmotic) or GYM medium (hypo-osmotic) supplemented with Dilworth's vitamins for 2 days, and the growth was compared with that of strains grown in TY medium. OD_600 _values were measured. Percentage growth values are the mean and SD from three independent trials.

† Difference between the wild type and the *rosR *mutants is statistically significant at P < 0.05 (Student's *t *test).

The *rosR *mutants were also significantly more sensitive to hyper- and hypo-osmotic stress than the wild type (Table 2). The mutants achieved only 10-12% of the growth in TY medium supplemented with 85 mM NaCl (the highest NaCl concentration tolerable by the wild type) when compared to a control medium without NaCl. The growth of the *rosR *mutants was also significantly diminished in relation to the wild type strain in hypo-osmotic GYM medium. The higher sensitivity of the *rosR *mutants to hypo-osmotic stress might be explained by an increased permeability of their cell membranes allowing greater amounts of neutral polysaccharide (e.g. β-glucan) to be excreted [34,35]. Taken together, *rosR *mutation seems to affect membrane integrity, resulting in an altered sensitivity to detergents, ethanol, and osmotic stresses.

### Changes in membrane and extracellular protein profiles of the *rosR *mutant in relation to the wild type

To examine the role of *rosR *in the putative membrane leakage, membrane and extracellular proteins of Rt2472 and Rt24.2 grown in TY medium were compared by SDS-PAGE (Figure 4B). Some differences in membrane protein profiles were observed, such as two abundant bands with an estimated mass of ~30 kDa and one band of ~63 kDa in Rt2472. In contrast, the amounts of proteins of ~20, 34, and 36 kDa were significantly diminished in this mutant. Based on the literature data, the masses of these three proteins corresponded to mature proteins RopB1 (20.1 kDa), RopA (36 kDA), and RopA1 (38 kDA), which had been identified in *R. leguminosarum *[36-38].

An extracellular protein profile of *R. leguminosarum *bv. *trifolii *24.2 was very similar to that of *R. leguminosarum *bv. *viciae *3841 [22]. In extracelullar protein profiles of Rt24.2 and Rt2472, besides several bands common to both supernatants, a protein of ~13 kDa was uniquely present, and a protein of ~83 kDa was significantly more abundant in the *rosR *mutant supernatant (Figure 4B). On the other hand, the amounts of proteins of about 36 kDa were drastic diminished in the Rt2472 culture supernatant. The differences in protein patterns between the wild type and the *rosR *mutant indicated that some additional proteins were being secreted from the cells, perhaps as a result of unspecific membrane leakage, possibly due to changes in membrane permeability triggered by the mutation.

To study the effect of clover root exudates on the protein profiles of Rt2472 and Rt24.2, the strains were cultured in M1 medium with or without 5 μM exudates, and membrane and extracellular proteins were isolated (Figure 4C). It was observed that this culture medium influenced both extracellular and membrane proteins when compared to TY grown cultures. Most apparent differences were found for secreted proteins. For Rt2472 and Rt24.2, proteins of about 60 kDa and 31 kDa (for Rt24.2 also a protein of ~35 kDa) present in TY supernatants were absent when these strains grew in M1. On the other hand, additional proteins were present in M1 supernatants. Some differences between the *rosR *mutant and the wild type were detected in the proteins from M1 supernatants. However, the effect of root exudates on extracellular protein profiles was not noticeable.

In membrane proteins, a major difference concerned two proteins of ~38 kDa and ~20 kDa, which were present in both strains grown in TY medium but were missing in the M1 grown cultures (Figure 4C). No visible differences in protein profiles were detected between these two strains grown in M1 and in the presence of root exudates.

The purity of the membrane and the extracellular protein fractions isolated from Rt2472 and Rt24.2 was assayed by Western blotting with anti-PssB and anti-PssN antisera specific to *R. leguminosarum *(see additional file 1: Figure S1). PssB, previously described as cytoplasmic inositol monophosphatase present in two forms of 32 and 29.5 kDa, was used as a marker of cytoplasmic proteins [39], and PssN lipoprotein (43-kDa) as a marker of membrane proteins [40]. No substantial contamination of membrane and extracellular protein fractions by this cytoplasmic protein was detected (Figure S1A). For PssN, besides a strong signal in membrane fractions, residual signals were also detected in the cytoplasmic fraction and extracellular proteins of Rt24.2 grown in M1 (Figure S1B). This finding was in agreement with the previously described detection of low amounts of PssN in the culture supernatant [40].

To identify the individual membrane and extracellular proteins of the *rosR *mutant that differed in abundance from those of the wild type strain, we submitted them to Edman degradation sequencing. Unfortunately, possibly due to blocked N-terminus of the proteins, only the protein of 20 kDa that was less abundant in the *rosR *mutant TY medium membrane fraction, was identified by this method. The sequence of the 15 N-terminal amino acids (ADAVDQVPEAPVAQD) showed 100% identity to the 25-39 aa region of the OmpA-like protein of *R. leguminosarum *bv. *trifolii *WSM1325 (C6AU25), the outer membrane protein RopB1 of *R. etli *CFN42 (Q2KA52), and RopB1 of *R. etli *CIAT652 (B3PV86).

### *R. leguminosarum *bv. *trifolii rosR *mutants are altered in motility and biofilm formation

**Figure 5:**
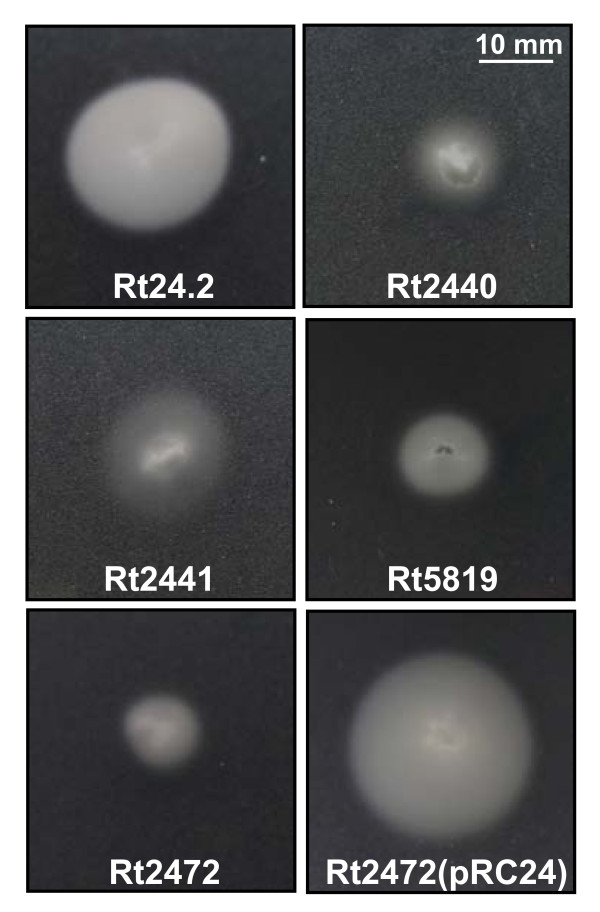
**Motility of *R. leguminosarum *bv. *trifolii *24.2 wild type and its derivatives after 3-day incubation at 28°C on 0.3% M1 agar plates**.

The effect of *rosR *mutation on the motility of *R. leguminosarum *was assessed (Figure 5) and a very strong inhibition of motility in the studied mutant strains was observed. The swimming zones were from 2- (Rt2441) to 2.5-fold smaller (Rt2440 and Rt2472) than for Rt24.2 wild type following growth on M1 semisolid medium for 72 h. The Rt5819 strain, entirely deficient in EPS synthesis due to a mutation in *pssA *encoding a glucosyl-IP-transferase, showed a similar motility-deficient phenotype. Complementation of the *rosR *mutation with pRC24 carrying wild type *rosR *fully restored the swimming radius of Rt2472. The results demonstrate that the *rosR *mutation negatively affected mutant motility.

**Figure 6:**
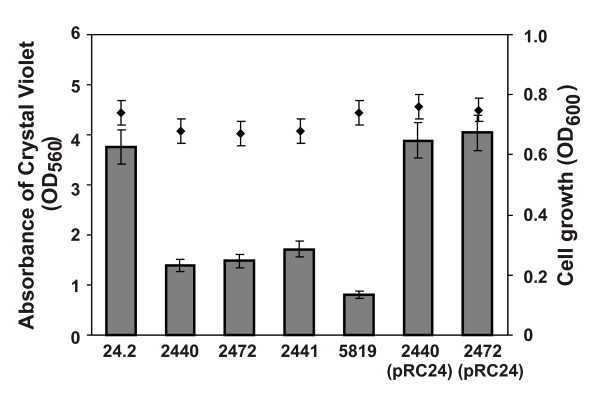
**Quantification of biofilm formation (bars) and bacterial growth (rombs) of *R. leguminosarum *bv. *trifolii *24.2 wild type and its derivatives measured after 48 h**. Data shown are the means of three replicates ± SD.

To determine whether the *rosR *mutation affected biofilm formation, growth of the wild type and the *rosR *mutants was analyzed in M1 in a microtiter plate assay. This medium was used in an attempt to reflect soil conditions where nutrients are usually scarce. In the assay, the mass of biofilm formed by the *rosR *mutants, as measured by crystal violet binding, was substantially lower, i.e., 37% (Rt2440) and 45% (Rt2441), respectively, in relation to the wild type (Figure 6). The *R. leguminosarum *bv. *trifolii pssA *mutant, included in this assay, formed only 18% of the wild type biofilm, which confirms the earlier observations on biofilm formation by an *R. leguminosarum *bv. *viciae pssA *mutant [14]. Complementation of *rosR *mutation with pRC24 restored biofilm development to the wild type levels (Figure 6).

**Figure 7:**
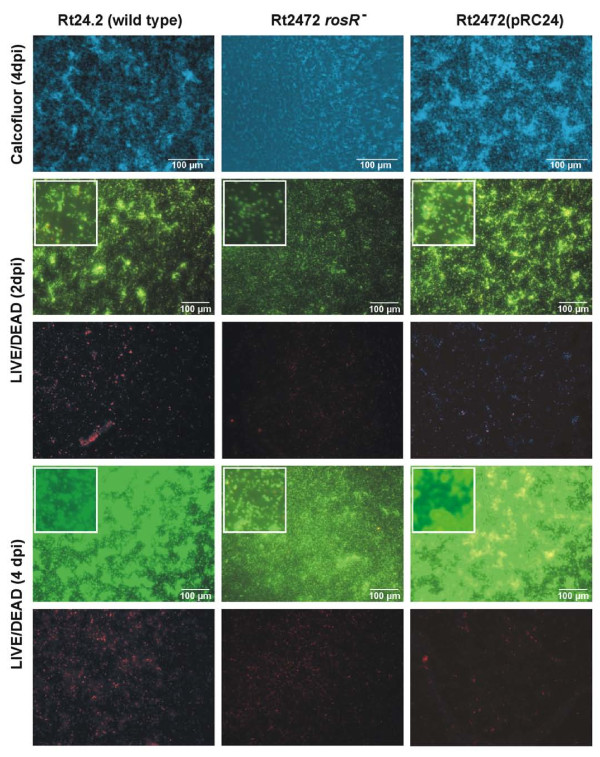
**Developing stages of biofilm formation in *R. leguminosarum *bv. *trifolii *wild type 24.2, *rosR *mutant Rt2472 and Rt2472(pRC24) strains observed after 2 and 4 days**. The *rosR *mutant Rt2472 did not form typical biofilm after 4 days and was restored to the wild type phenotype after introduction of the *rosR *gene cloned on pRC24 plasmid. Top panel shows 4 dpi biofilms stained with Calcofluor, and the remaining panels show horizontal projected images from 2 and 4 dpi biofilms, with live (Syto-9, green fluorescence) and dead (propidium iodide, red fluorescence) cells. The insets show details of individual stages of biofilm formation.

The *rosR *mutant (Rt2472) and the wild type strain were chosen to examine the organization and viability of *R. leguminosarum *bv. *trifolii *cells in biofilm. The organization of adherent bacteria on plastic surfaces differed substantially between the wild type and the mutant (Figure 7). After four days of growth, the Rt24.2 formed a typical mature biofilm with water channels. The parameters describing the biofilms formed by the wild type and the *rosR *mutant are listed in Table 3. The *rosR *mutant developed a biofilm which was nearly two times thinner than the wild type's, and which was unorganized and impaired in maturation, with a significantly lower number of viable cells. Complementation of the *rosR *mutant with pRC24 restored the wild type phenotype, and Rt2472(pRC24) formed a mature biofilm with highly viable bacteria, comparable to the wild type (Figure 7).

**Table 3:** The parameters of biofilms formed by the *R. leguminosarum *bv. *trifolii *wild type and Rt2472 *rosR *mutant.

Strain	Ratio of live/dead cells	Depth of biofilm (μm)	Area covered by biofilm (%)	Fractal dimension (scalar units)	Outline (×10^3^) (μm)
Rt24.2	51.06 ± 6.12	47.33 ± 1.15	87.57 ± 6.36	1.425 ± 0.05	109.25 ± 5.9
Rt2472	27.53 ± 4.57†	25.66 ± 1.52†	50.17 ± 5.08†	1.325 ± 0.14	69.71 ± 1.2†
Rt2472(pRC24)	71.86 ± 3.07	54.26 ± 3.94	88.82 ± 8.78	1.417 ± 0.06	113.57 ± 10.8

† Difference between the wild type and the *rosR *mutant is statistically significant at P < 0.05 (Student's *t *test).

### Effect of clover root exudates on growth of *rosR *mutants and EPS production

**Figure 8:**
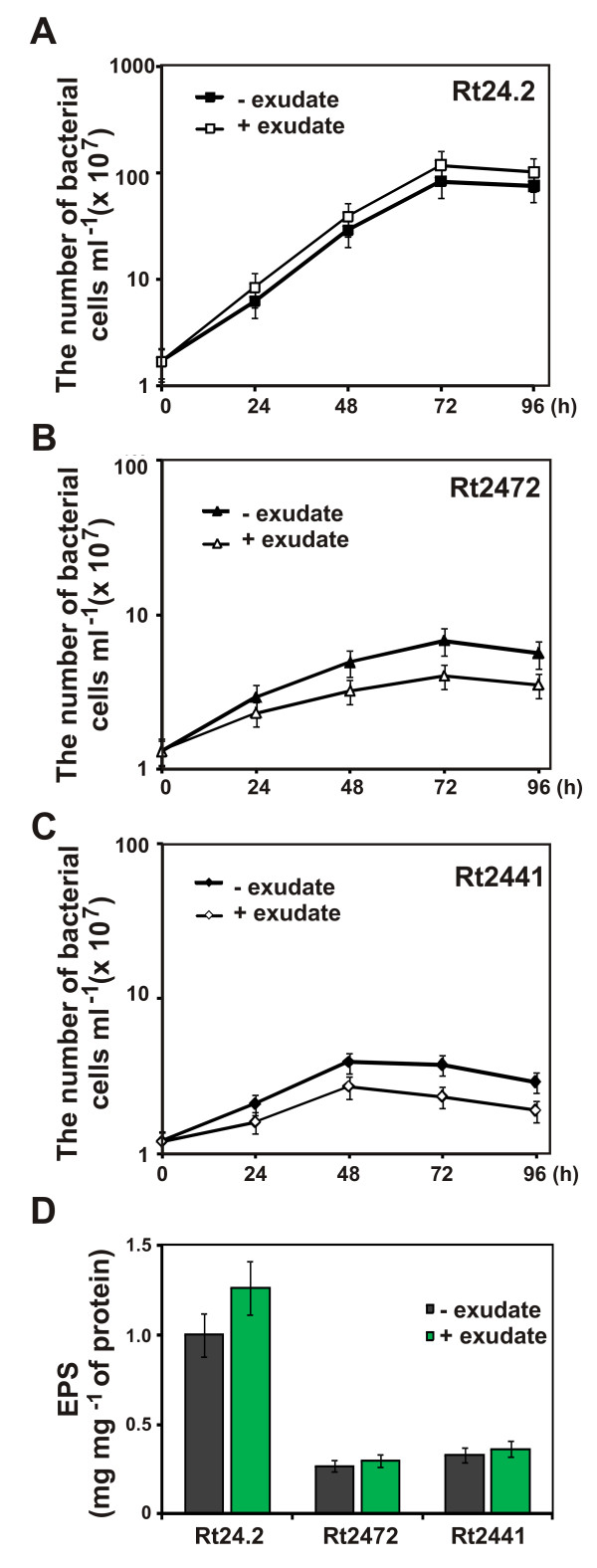
**The effect of clover root exudates on the growth of Rt24.2 wild type (A), and Rt2472 (B) and Rt2441 (C) *rosR *mutants. (D) The effect of clover root exudates on the EPS production by the wild type and the *rosR *mutants**. Data shown are the means of three replicates ± SD.

The increased sensitivity of the *rosR *mutants to surface active compounds (Table 2) and some antibiotics, most probably caused by changes in membrane protein profiles (Figure 4), inclined us to assess the effect of clover root exudates on growth of the *rosR *mutants. The strains were grown in M1 medium supplemented with 5 μM root exudates, and aliquots of the cultures were plated in dilutions on 79CA medium. Clover root exudates slightly enhanced the growth of the Rt24.2 wild type (Figure 8A). The *rosR *mutants (Rt2472 and Rt2441) grew significantly slower than the wild type in M1 medium and were more sensitive to the root exudates (Figure 8B-C). In the presence of the exudates, Rt24.2 produced a significantly increased amount of EPS, whereas the level of EPS produced by the *rosR *mutants was increased only slightly (Figure 8D).

### Phenotype analysis of a *rosR *mutant using Biolog tests

In several experiments, we noticed that the *rosR *mutants grew slower than the wild type both in liquid and solid media, suggesting changes in their metabolic capabilities. In an attempt to define the phenotype profile of the *rosR *mutant (Rt2472) in relation to the wild type strain, the PM system (Biolog) was used [41]. PM1, PM2A, PM3B, and PM4A plates were chosen, allowing for examination of the utilization of 190 different carbon sources and 95 nitrogen, 59 phosphorus, and 35 sulfur sources. In addition, PM9 plates were used to test the growth under various stress conditions.

**Figure 9:**
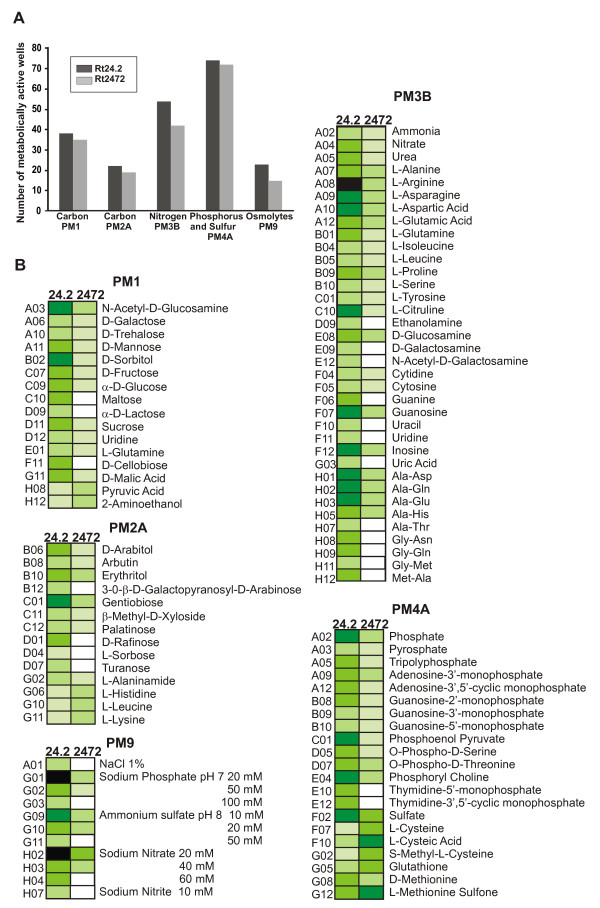
**A quantitative and qualitative comparison of the carbon, nitrogen, phosphorus, and sulfur sources metabolized by the *rosR* mutant and the wild type strain.** (A) The number of metabolized compounds by the *rosR* mutant Rt2472. (B) Metabolic differences between the wild type Rt24.2 and Rt2472 mutant in PMs. The following color code for the level of utilization of metabolic sources is used: OD_600 _<0.1, very light green; OD_600 _between 0.1 and 0.2, light green; OD_600 _between 0.2 and 0.3, medium green; OD_600 _between 0.3 and 0.4, dark green; OD_600 _> 0.4, black; unutilized metabolites are denoted by white boxes. Data shown are the means of two replicate experiments.

In general, the *rosR *mutant utilized fewer energy sources and was significantly more sensitive to the majority of the tested osmolytes than the wild type (Figure 9A). The most visible differences were observed in utilization of carbon and nitrogen sources (Figure 9B). Mutant Rt2472 utilized several carbon and nitrogen sources two to four times less efficiently than the parental strain. In contrast, utilization of some amino acids, pyruvic acid, and 2-aminoethanol (PM2A) by the *rosR *mutant was considerably higher than for the wild type. Moreover, nine of the tested sugar sources and twelve of the nitrogen sources were not utilized by the *rosR *mutant (PM1, PM2A, and PM3B) (Figure 9B).

The phenotype of the Rt2472 mutant did not differ essentially from the wild type with regard to utilization of phosphorus sources (PM4B) except that they were metabolized less effectively. It is worth noting that the Rt2472 significantly better utilized sulfur sources, such as L-cysteine, L-cysteic acid, and S-methyl-L-cysteine (PM4A), than the wild type. This suggests derepression of the sulfur metabolic pathway in the *rosR *mutant background.

PM9 microplates were used to determine the sensitivity of the *rosR *mutant to several osmolytes. We observed a significant increase in *rosR *mutant sensitivity in the presence of NaCl, Na_3_PO_4_, (NH_4_)_2_SO_4_, and NaNO_3_. In contrast to the wild type, Rt2472 did not survive in 100 mM Na_3_PO_4_, 50 mM (NH_4_)_2_SO_4_, 60 mM NaNO_3_, and 10 mM NaNO_2 _(Figure 9B).

In summary, the *rosR *mutant was impaired in its ability to utilize several compounds and exhibited an increased sensitivity to some osmolytes, suggesting a role of RosR protein in the control of many essential metabolic processes.

### Effect of *rosR *mutation on root hair attachment and infection

**Figure 10:**
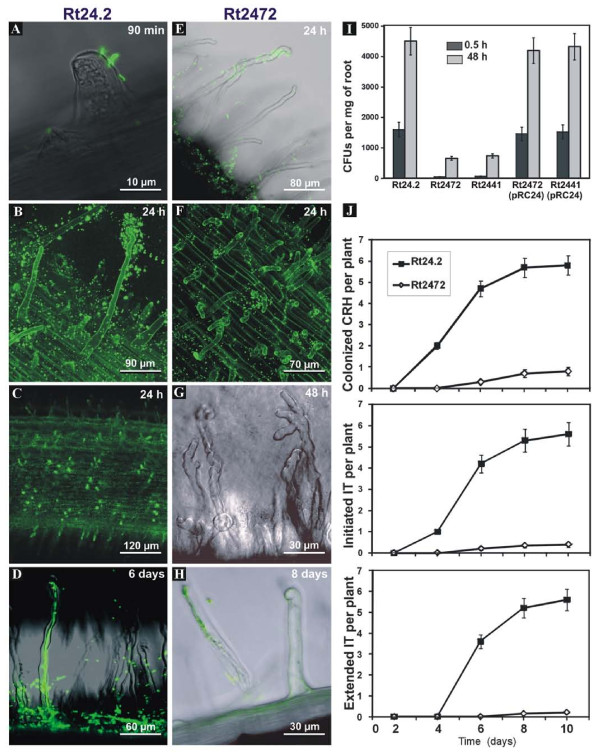
**Root attachment and infection of clover roots by the *rosR *mutant and the wild type**. Fluorescence microscopy analyses of clover root colonization and invasion by GFP-expressing cells of *R. leguminosarum *bv. *trifolii *wild type (A-D) and the *rosR *mutant (Rt2472) (E-H). The Rt24.2 cells attached very fast and effectively to root hairs (A-B), and formed caps on the top of root hairs (C). (D) Curled root hairs with an extended infection thread filled with the wild type cells. The infection thread started from the Shepherd's crook of the curled root hair and reached the base of root hair. The ability of root attachment and root cap formation of the *rosR *mutant was substantially decreased (E-F). Only individual cells of the Rt2472 *rosR *mutant attached to root hairs (E) and root caps were formed sporadically (F). Several root hairs showed abnormal deformation (G). The root hair colonized by the *rosR *mutant, which had developed an aborted infection thread (H). (I) Attachment to clover roots 0.5 h and 48 h post inoculation with the wild type, and the Rt2472 and Rt2441 *rosR *mutants, and their derivatives complemented with pRC24. For each strain, ten roots were examined. Data shown are the means of two replicates ± SD. (J) Kinetics of curled root hair (CRH) formation, infection thread (IT) initiation and extension on clover plants inoculated with the wild type and the *rosR *mutant (Rt2472). For each strain, 25 plants were used. Data shown are the means of two experiments.

The *rosR *mutants formed significantly fewer nodules on clover roots than the wild type strain and their appearance was delayed (Table 1). This might indicate a failure in the first stages of mutant strain's interaction with the roots. To visualize root hair attachment of rhizobia and their ability to grow on the root surface and infect root hairs, the Rt24.2 and Rt2472 strains harbouring plasmid pHC60 with constitutively expressed *gfp *[42] were used. Clover seedlings inoculated with the bacteria on Fåhraeus medium-covered microscope slides were observed in the course of time. After the first 90 min, single Rt24.2 cells were visible on the surface of root hairs (Figure 10A). After 24 h, attachment of numerous Rt24.2 cells to root hairs was seen. Bacteria were located mainly on root hair tops, forming caps and rhizobial clouds (Figure 10B). In the zone of growing root hairs, the majority of the root hairs were coated with Rt24.2 cells (Figure 10C). After 6 days post infection (dpi), infection threads inside some of the root hairs were initiated or already extended and reached root epidermal cells (Figure 10D). In contrast, Rt2472 cells were seen on the root surface but were attached to the root hairs only sporadically demonstrating a much weaker attachment ability (Figure 10E). The caps formed by *rosR *cells on the top of root hairs were detected very rarely (Figure 10F). In addition, several root hairs had an atypical, expanded shape resembling ginger roots (Figure 10G) in contrast to the typical curled root hairs in clover inoculated with the wild type. In the case of *rosR *mutant-inoculated plants, infection threads inside root hairs were observed sporadically, and their elongation was frequently interrupted (Figure 10H).

To quantitatively determine the attachment ability to the surface of clover roots, Rt24.2 wild type, Rt2472 and Rt2441 *rosR *mutants, and their derivatives bearing plasmid pRC24 were incubated with clover roots for 0.5 h and 48 h. The wild type cells showed a high attachment ability to the root surface (Figure 10I). In contrast, the number of Rt2472 and Rt2441 cells attached to roots during 0.5 h was drastically lower (3.6% and 4.7% of the wild type, respectively). After 48 h, the *rosR *mutant cells were still considerably less numerous than Rt24.2 (14.6% for Rt2472 and 16.5% for Rt2441). These assays confirmed that *rosR *mutation affects the first step of the infection process, i.e., bacterial adhesion to root hairs (Figure 10I).

To study the further stages of clover infection, seedlings were inoculated with Rt24.2 and Rt2472 tagged with *gfp *and observed under a light microscope during a 10-day experiment. The following were quantified: (i) tightly curled root hairs containing trapped rhizobia, (ii) initiated (immature or aborted) infection threads, and (iii) infection threads which successfully entered the root cortex of clover. As was shown in Figure 10J, wild type bacteria effectively colonized curled root hairs, and the first initiated infection threads were observed after 4 dpi. Extended infection threads were formed from almost all colonized root hairs, giving, on average, 5.6 successful infections per plant after 10 days. The *rosR *mutant exhibited notable differences in infection thread formation. Rt2472 cells colonized root hairs very rarely and with a delay in comparison to the wild type. As a consequence, the initiation of infection threads was observed only occasionally and a great majority of the infection threads was not properly extended and did not reach root cortical cells (Figure 10J).

## Discussion

In this paper, we present data showing that RosR of *R. leguminosarum *bv. *trifolii *24.2, besides its role in transcriptional regulation of EPS synthesis, is required for successful interaction with clover plants, stress tolerance, motility, and biofilm formation. Both the *rosR *mutants (Rt2440 and Rt2472) described earlier [23,30] and the newly isolated Rt2441, bearing a genomic wild type *rosR *with the regulatory region in addition to the mutated *rosR *copy, displayed pleiotropic phenotypes. Pleiotropy of the *rosR *mutants was fully restored in complementation tests using a low-copy plasmid carrying *rosR*. Interestingly, the Rt2441 mutant showed a negative dominant effect on EPS production, which confirmed the regulatory role of RosR in EPS synthesis. This phenomenon could be explained, to some extent, by negative autoregulation of *rosR *expression [23], which may be strengthened by the presence of more RosR-boxes binding RosR (Figure 2). As a result, the diminished amount of functional RosR might be insufficient for positive regulation of EPS production. The negative dominance could be overcome by introducing additional copies of *rosR *in the complementation experiments (Table 1, Figure 2). A similar dominant-negative effect of *rosAR *mutation in *A. radiobacter *had been described by Brightwell et al. [43]. The introduction of *rosAR *lacking the 3" terminus on a plasmid into a wild type strain resulted in non-mucoid transconjugants.

All the *rosR *mutants were considerably impaired in both the level of EPS production and the rate of its polymerization. They produced three times less EPS which was also slightly changed in non-carbohydrate modification and the level of polymerization. In addition, PS part of Rt2440 LPS showed quantitative differences in the sugar composition (mainly in 6-deoxysugars ratio) in comparison to the wild type PS.

Like most *R. leguminosarum *bv. *trifolii *mutants deficient in surface polysaccharide production [6], the *rosR *mutants elicited nodules in which rhizobia did not fix nitrogen. These mutants were also not competitive in relation to the wild type. Rt2472 and Rt2441, even when present in the inoculum in 1000-fold excess to the wild type, occupied only about 10% of the clover nodules. An *R. etli rosR *mutant formed colonies with an altered morphology, but retained the ability to elicit nitrogen-fixing nodules on *Phaseolus vulgaris*, which forms determinate-type nodules [24]. Nevertheless, the nodulation competitiveness of that *rosR *mutant was greatly reduced and, for this reason, *rosR *was considered a determinant of *R. etli *competitiveness.

One of the most striking effects of *rosR *mutation in *R. leguminosarum *bv. *trifolii *is the drastic decrease in attachment to root hairs and growth on the root surface. In contrast to the wild type strain, *rosR *mutant cells only sporadically formed caps on the top of root hairs, and, consequently, infection threads were initiated rarely, and the majority of them were aborted. Recently, a similar effect of *R. leguminosarum pssA *mutation has been described: the mutant was defective in attachment and biofilm formation both *in vitro *and on root hairs [18]. An *R. leguminosarum gmsA *mutant, which did not produce glucomannan, demonstrated a very similar symbiotic phenotype to the *rosR *mutant Rt2472. It was defective in attachment and biofilm formation on root hairs and was strongly outcompeted by the wild type in mixed inoculations, showing that glucomannan is critical for competitive nodulation [18]. In the case of *R. leguminosarum *cellulose synthesis mutant (*celA*) only individual cells attached to root hairs, but caps were not formed [18]. Other EPS-deficient mutants such as *R. leguminosarum (pssD) *and *S. meliloti *(*exoY*) were defective in infection thread formation [42,44]. In *S. meliloti*, an *exoH *mutant lacking the succinyl modification in succinoglycan and an *exoZ *mutant producing this heteropolymer without the acetyl modification exhibited a reduced efficiency in the initiation and elongation of infection threads [42]. *S. meliloti exoR *and *exoS *mutants overproducing EPS I demonstrated a marked reduction in the biosynthesis of flagella resulting in a loss of the ability of the cells to swarm and swim and had a significantly reduced efficiency of root hair colonization [45]. In conclusion, defective attachment and infection thread formation in the first stages of symbiosis seem to be common features of rhizobial mutants which produce altered surface polysaccharides and infect plants which form indeterminate-type nodules.

The pleiotropic effect of *rosR *mutation was also expressed as an increased sensitivity to detergents, hyper- and hypo-osmotic stress, and antibiotics from the beta-lactam group which affect peptidoglycan synthesis. The Rt2472 mutant also exhibited an increased sensitivity to several osmolytes indicating that RosR is engaged in the regulation of many essential cell processes. These changes in the phenotype indicated a direct or indirect effect of *rosR *mutation, which, presumably, affects membrane integrity or causes outer membrane instability. This was partially evidenced by SDS-PAGE of membrane and secreted proteins isolated from the wild type and *rosR *mutant (Rt2472). We observed some differences in the protein profiles of both strains, especially when they were cultured on TY rich medium. Out of the several membrane proteins whose concentrations were significantly decreased in the *rosR *mutant, three proteins corresponded to outer membrane proteins RopB1 (20.1 kDa), RopA (36 kDA), and RopA1 (38 kDA) of *R. leguminosarum *[36-38]. Among them, the 20 kDa protein was identified as OmpA-like RopB1. The diminished amount of this protein in the *rosR *mutant could influence its membrane integrity and sensitivity to surface-active compounds and some antibiotics. Several classes of outer membrane proteins (OMPs) of *R. leguminosarum *bv. *viciae *strain 248 had been described as antigens, and the level of some of them significantly decreased during bacteroid differentiation [36-38]. Recently, a gene family of OMPs (*ropB*, *ropB2*, and *ropB3*) in *R. leguminosarum *bv. *viciae *VF39SM has been described [46]. A *ropB *mutant was characterized by an increased sensitivity to detergents, hydrophobic antibiotics, and weak organic acids, which suggested a role of RopB in outer membrane stability [46].

Extracellular protein profile of *R. leguminosarum *bv. *trifolii *24.2 wild type growing in TY was very similar to that of *R. leguminosarum *bv. *viciae *3841 described by Krehenbrink and Downie [22]. Significant differences between TY supernatant protein profiles of the Rt24.2 and the Rt2472 were observed. The main difference was essentially diminished the amount of proteins of about 35 kDa in the *rosR *mutant. In the supernatant of *R. leguminosarum *bv. *viciae *3841, proteins of similar molecular masses (35.6-kDa Leu/Ile/Val-binding protein, 34.1-kDa flagellin, and 34.1-kDa basic membrane lipoprotein) were identified.

Moreover, extracellular proteins of the wild type and the *rosR *mutant differed depending on growth in complex (TY) or minimal (M1) media, similarly to proteins secreted by the *R. leguminosarum *bv. *viciae *3841 *prsD *mutant [22].

*R. leguminosarum *bv. *viciae *3841, an LPS mutant (*fabF2/F1*) lacking the very long chain fatty acid (27OHC_28:0_) component of lipid A, displayed an increased sensitivity to membrane stressors and an increase in the secretion of neutral surface polysaccharides, namely, periplasmic cyclic β-(1,2)-glucans [35]. The production of these compounds is associated with hypo-osmotic stress tolerance in rhizobia [47]. The higher sensitivity of the *rosR *mutants to hypo-osmotic stress might be explained by increased permeability of their cell envelopes, which could allow excretion of greater amounts of neutral polysaccharides.

Recently, several other osmotically unstable rhizobial mutants have been described, among them salt-sensitive mutants of *S. meliloti*, some of them significantly affected in competing against the wild type for nodule occupancy [48]. Mutation in *S. meliloti *regulatory gene *nesR *affected competition for nodulation, adaptation to high osmolarity, and nutrient starvation [49]. Also, genes encoding trehalose biosynthesis pathways and potassium uptake systems were found to be important for *S. meliloti *growth in hyperosmotic medium [50,51].

*R. leguminosarum *bv. *trifolii rosR *mutants deficient in EPS production grew considerably slower than the wild type on minimal medium. Using the Biolog system, we established that the *rosR *mutant revealed differences in utilization of carbon and nitrogen sources in relation to the wild type. Similarly, phenotypic analysis of *S. meliloti exoS *and *chvI *null mutants demonstrated that ExoS/ChvI regulatory system not only controls succinoglycan (EPS I) and galactoglucan (EPS II) synthesis but is also required for growth on over 21 different carbon sources [52]. The *chvI *mutant exhibited several pleiotropic effects: failed to grow on complex medium, had an altered LPS profile, exhibited lower tolerance to acidic conditions, was hypermotile, and synthesized significantly less poly-3-hydroxybutyrate than wild type, indicating that ChvI is engaged in regulatory networks involving the cell envelope and metabolism [53].

In several studies, a connection between the production of bacterial polysaccharides and motility has been established. Both *R. leguminosarum *bv. *trifolii rosR *mutants and the *pssA *mutant deficient in EPS production exhibited a significant decrease in motility. *S. meliloti *MucR protein that simultaneously acts as a transcriptional repressor of galactoglucan synthesis and an activator of succinoglycan synthesis [25,54] inhibits the expression of *rem *encoding an activator of the expression of such genes as *flaF *and *flgG *[55]. Other regulatory proteins, such as the ExpR/Sin quorum system, are additionally engaged in the regulation of *S. meliloti *motility [56,57]. A non-motile phenotype has also been described for *ndvA *and *ndvB *mutants defective in the synthesis of β-(1,2)-glucans under hypo-osmotic conditions [58,59]. Alterations in the LPS structure often cause motility-related defects [60,61]. The *R. leguminosarum *bv. *viciae *3841 LPS mutant mentioned earlier was impaired in motility and biofilm formation. In this mutant, the motility genes *flaA, mcpC, mcpD, visN*, and *rem *were significantly down regulated when compared with the wild type [35].

The *R. leguminosarum *bv. *trifolii rosR *mutants formed significantly reduced amounts of biofilm, which was altered in structure and maturation and contained more dead cells in comparison to the wild type. The Rt24.2 *pssA *mutant formed smaller amounts of biofilm in comparison to the *rosR *mutants, which confirms the important role of this polymer in biofilm development. Similarly, *R. leguminosarum *bv. *viciae pssA *mutant was unable to develop microcolonies and more complex biofilm structures [14,18]. The presence of a RosR-box motif in the promoter region of *R. leguminosarum *bv. *trifolii pssA *and the significantly lower expression of *pssA-lacZ *fusion in the *rosR *mutant than in the wild type indicate positive regulation of this gene by RosR [23,62]. In *S. meliloti*, the LMW fraction of EPS II was established to be crucial for formation of a biofilm with a highly ordered structure [15,16]. EPS II non-producing strains or those producing only the HMW fraction of this polysaccharide formed very low amounts of biofilm [15]. In the case of Rt2440 and Rt2441, the amount of LMW EPS was diminished, but the role of this fraction in biofilm formation remains to be elucidated. Beside rhizobial surface components, such as EPS and LPS, and quorum sensing systems, several other environmental factors affect biofilm formation, among them catabolite repression and nutrient limitation [63-65].

## Conclusions

In the present study, we characterized *rosR *mutants bearing a mutation in the gene encoding a transcriptional regulator with a C_2_H_2 _type zinc-finger motif. We demonstrated the importance of the intact *rosR *gene both in the interaction with the host plant and in the bacterial adaptation to stress conditions. The pleiotropic effects of the *rosR *mutation confirmed the importance of this gene not only for exopolysaccharide production, but also for several other metabolic traits.

## Methods

### Bacterial strains, plasmids, and growth conditions

Bacterial strains, plasmids, and oligonucleotide primers used in this study are listed in Table 4. *R. leguminosarum *strains were grown in 79CA with 1% glycerol as a carbon source [66] and tryptone-yeast (TY) complex media, or M1 minimal medium [67] containing 1% glycerol and Dilworth's vitamins [68] at 28°C. *E. coli *strains were grown in Luria-Bertani (LB) medium at 37°C [67]. Where required, antibiotics for *E. coli *and *R. leguminosarum *were used at the following final concentrations: kanamycin, 40 μg/ml; rifampicin 40 μg/ml; ampicillin, 100 μg/ml; tetracycline 10 μg/ml; and nalidixic acid, 40 μg/ml.

**Table 4:** Bacterial strains, plasmids, and primers used in this study.

Strain, plasmid or oligonucleotide primers	Relevant characteristics	Reference
***R. leguminosarum ***bv. *trifolii *24.2	Wild type, Rif^r^, Nx^r^	[23]
Rt2440	Rt24.2 derivative carrying *rosR *with one nucleotide deletion (ΔC177)	[23]
Rt5819	Rt24.2 derivative carrying the mini-Tn*5 *between 363-364 bp position of *pssA*	[30]
Rt2472	Rt24.2 derivative carrying the mini-Tn*5 *between 151-152 bp position of *rosR*	[30]
Rt2441	Rt24.2 with additional *rosR *upstream region introduced by pM41 integration, Km^r^, Nx^r^	This work
***E. coli***		
DH5α	*supE*44 Δ*lac*U169 (φ80 *lacZ*Δ M15) *hsdR*17 *recA*1*endA*1*gyrA*96 *thi*-1 *relA*1	[67]
S17-1	294 derivative RP4-2Tc::Mu-Km::Tn7 chromosomally integrated	[79]
**Plasmids**		
pK19mobGII	*mob, lacZ*α, *gusA*, Km^r^	[80]
pBBR1MCS-2	*mob, lacZ*α, Km^r^	[81]
pB31	pUC19 with 1174-bp *Bam*HI fragment containing Rt24.2 *rosR*	[23]
pM41	pK19mobGII with 586-bp *Eco*RI-*Pst*I fragment from pB31 containing the *rosR *upstream region	This work
pRC24	pRK7813 with 1174-bp *Bam*HI fragment containing *rosR *of Rt24.2	[23]
pBR24	pBBR1MCS-5 with 1174-bp *Bam*HI fragment containing *rosR *of Rt24.2	[23]
pEX1	pBBR1MCS-2 with 586-bp *Eco*RI-*Pst*I fragment containing the upstream region and the first 60 codons for RosR	This work
pEX8	pBBR1MCS-2 with 372-bp *Eco*RI-*Xba*I fragment containing the -403 bp to -32 bp *rosR *upstream region	This work
pEX9	pBBR1MCS-2 with 219-bp *Eco*RI-*Xba*I fragment containing the -403 bp to -185 bp *rosR *upstream region	This work
pEX60	pBBR1MCS-2 with 278-bp (-96 bp to +182 bp) *Eco*RI-*Pst*I fragment containing the first 60 codons for RosR cloned downstream the vector promoter	This work
pBR28	pBBR1MCS-2 with 820-bp (-96 bp to +724 bp) *Eco*RI-*Bam*HI fragment containing the full-length *rosR *cloned downstream the vector promoter	This work
pHC60	Vector with *gfp *and RK2 stabilization fragment, Tc^r^	[39]
**Oligonucleotide primers **	Sequence (5'-3')^*^	
pEP1	ATGCAAGAATTCTGCACAGGAAGC	[23]
pEP5	CGGTCAGGAATTCTAAGAACAGGT	[23]
pEP6	TCGAAACAGGAATTCGATTCCTGC	[23]
pRR1	CGCATTCTAGACATGTGATCTGCT	[23]
pEP8	AACGGCTCTAGACTGACACGCCAAA	[23]
pEP9	TCATGCTCTAGACGATGGCCTCAGT	[23]
rosA	GCGGATCCGCGACTTTACCAGATTTA	[23]
rosB	GTCACGCTCTTCGGAATTCAGGGGT	[23]
rosC	AGGGATCCATTCTAAACCTGTCGGCA	[23]
rosD	TCGGATCCTGTCGGCAAAGCATAAGA	[23]
rosG1	GACGATCGAATTCGGCCGTCTCTT	This work
rosD4	TTGCGGATCCGCAGATGCCGGT	This work
rosD5	ACCACGCCTGGGATCCAGGAAAA	This work

* Sequences for *Eco*RI, *Bam*HI and *Xba*I restriction sites are underlined.

To assay the effect of clover root exudates on growth of the *rosR *mutants (Rt2441 and Rt2472) and the wild type, the strains were grown in 5 ml M1 medium supplemented with 5 μM exudates, which was prepared as described previously [69]. After 24, 48, 72, and 96 h, 100 μl aliquots of each culture were removed and plated in dilutions on 79CA plates, incubated 4 days at 28°C, and the colonies were counted.

### DNA methods: construction of Rt2441 *rosR *mutant and plasmids containing different fragments of the *rosR *upstream region and *rosR *ORF

Standard techniques were used for DNA isolation, restriction enzyme digestion, cloning, and Southern hybridization [67]. For PCR amplifications, Ready *Taq *PCR Reaction Mix (Sigma) or *Pfu*I polymerase (Fermentas) was used. Sequencing was performed using the BigDye terminator cycle sequencing kit (Applied Biosystems) and the ABI Prism 310 sequencer.

To construct Rt2441 mutant with a genomic insertion of an additional copy of *rosR *promoter region, 1.17-kb fragment containing the entire promoter region and 5'-end of *rosR *with *Pst*I internal restriction site was amplified using pB31 as a template and pEP1 and rosD primers. This amplicon was digested with *Eco*RI and *Pst*I and cloned into respective sites of suicide integrative pK19mobGII vector, giving pM41. The obtained construct was verified by sequencing. The pM41 was introduced into *E. coli *S17-1 by transformation, and then transferred from *E. coli *S17-1 into *R. leguminosarum *bv. *trifolii *24.2 via biparental conjugation. The transconjugants were selected on 79CA medium supplemented with nalidixic acid and kanamycin. The selected mutant was named Rt2441, and the insertion site was identified by PCR amplification (using primer sets: rosA/rosD, rosB/rosC, pEP1/pRR1, pEP5/pRR1, rosG1/pRR1, rosA/rosD4, rosB/rosD5), and Southern hybridization with a probe amplified on pB31 as a template and pEP1 and rosD primers.

To construct a set of plasmids containing different fragments of the *rosR *upstream region, the following primer pairs were used: pEP1/pRR1, pEP1/pEP8, pEP1/pEP9, pEP6/pRR1 and pEP6/rosD. Genomic Rt24.2 DNA was used as a template, yielding 586 bp, 372 bp, 219 bp, 278 bp, and 820 bp long amplicons. These PCR products were digested with: *Eco*RI and *Pst*I enzymes (586 bp and 278 bp fragments), *Eco*RI and *Xba*I (372 bp and 219 bp fragments) or *Eco*RI and *Bam*HI (fragment 820 bp), and cloned into respective sites of pBBR1MCS-2 vector, giving plasmids pEX1, pEX60, pEX8, pEX9 and pBR28, respectively. The obtained constructs were introduced by transformation into *E. coli *S17-1, and then transferred into *R. leguminosarum *bv. *trifolii *24.2 via biparental conjugation. The transconjugants were selected on 79CA medium supplemented with nalidixic acid and kanamycin.

### Phenotype analysis of *rosR *mutant using PM (Biolog) test

To compare a phenotype of the *rosR *mutant (Rt2472) with the wild type strain (Rt24.2), PM (Phenotype MicroArrays™, Biolog, USA) microplates PM1, PM2A, PM3B, PM4A and PM9 were used, according to manufacturer's instruction. Utilization of different carbon and energy sources by the strains was assayed using PM1 and PM2A microplates (190 compounds, including sugars and organic acids). PM3B plates were used for an examination of utilization of nitrogen sources (95 compounds), and PM4A plates of phosphorus and sulfur sources (94 compounds), accordingly. To test rhizobial growth under various stress conditions, PM9 plates were used. Rt2472 and Rt24.2 strains growing 48 h at 28°C on 79CA agar medium were collected and washed twice with sterile water. Final suspensions (OD_600 _of 0.1) were prepared in sterile IF-0a fluid supplemented with Dilworth's vitamins, and 100 μl aliquots were inoculated into microplate wells, and incubated at 28°C up to 72 h. For PM3B and PM4A plates, 1% glycerol as a carbon source and 20 μM FeCl_3 _were additionally added. Changes of color levels in the wells were monitored at the OD_595 _at regular time intervals using the Benchmark Plus™ microplate reader (Bio-Rad Laboratories, USA). The experiment was repeated twice.

### Assays for sensitivity to antibiotics, detergents, and osmotic stress

The sensitivity of *R. leguminosarum *bv. *trifolii *strains to sodium deoxycholate (DOC), sodium dodecyl sulfate (SDS), and ethanol was studied, and minimal inhibitory concentration of particular agents was determined. Bacteria were collected from TY agar medium into sterile water to an OD_600 _of 0.3 and 10 μl of each suspension was plated on TY containing a defined concentration of DOC (0.005-1% w/v), SDS (0.005-1% w/v) or ethanol (0.25-6% v/v). After 3 days, the growth of strains on individual media was determined. Three independent experiments were done for each strain.

To assess the effect of osmolarity on growth of the *R. leguminosarum *bv. *trifolii *Rt24.2 and the *rosR *mutants, the strains were grown in TY medium supplemented with a defined concentration of NaCl (0-510 mM). Cultures were incubated at 28°C for 48 h, and then the OD_600 _was measured. Tolerance to hypo-osmotic stress was determined using low-osmolarity glutamate-yeast extract-mannitol (GYM) medium [35]. Antibiotic sensitivity assays were performed using commercially available filter disks with the appropriate antibiotic: ampicillin (10 μg), erythromycin (15 μg), chloramphenicol (30 μg), gentamicin (10 μg), bacitracin (10 μg), augmentin (30 μg), streptomycin (10 μg), polymyxin B (10 μg), carbenicillin (20 μg), penicillin G (10 U), and tetracycline (30 μg) (Mast Diagnostics, Merseyside, UK). Filter disks were placed on the surface of 79CA medium, where 100 μl of *R. leguminosarum *cultures were previously spread. The diameter of the growth inhibition zone was measured after 3 days of incubation.

### Isolation and analysis of extracellular and membrane proteins

For analysis of extracellular and membrane proteins, the Rt2472 and Rt24.2 strains were grown at 28°C for 2 days to an OD_600 _of 0.6 in 200 ml TY medium. To study the influence of clover root exudates on membrane protein profiles, these strains were grown at 28°C for 3 days in 400 ml M1 medium supplemented with Dilworth's vitamins and with or without 5 μM exudates. Cells were removed by twice centrifugation at 5,000 × g for 20 min at 4°C, and supernatants were used for purification of extracellular proteins. The proteins were concentrated by precipitation with 10% trichloroacetic acid according to the procedure by Russo et al. [14]. Membrane proteins from cell pellets were isolated according to the method described by Kucharczyk et al. [70]. The cells were washed in 200 ml 50 mM Tris-HCl (pH 7.4), and centrifuged at 5,000 × *g *for 20 min at 4°C. Cell pellet was resuspended in 1.6 ml 200 mM Tris-HCl (pH 8.0), and then 1.6 ml 1 M sucrose in 200 mM Tris-HCl (pH 8.0), 16 μl lysozyme (12 mg/ml in 100 mM EDTA, pH 8.0) and 3.2 ml ice cold water were added. Next, 25.6 μl saturated ethanol-phenylmethylsulfonylfluoride (PMSF) solution and 12.8 μl 1 M dithiotreitol (DTT) were added, and probes were left on ice for 10 min. The cells were disrupted by sonication (8 × 10 s, 30 s breaks on ice, 50%) using the Misonix XL 2929 Sonicator Ultrasonic Processor with Cabinet (Misonix, Farmingdale, NY, USA). Unbroken cells were removed by centrifugation at 5,000 × *g *for 20 min. Supernatant was collected and transferred on the top of two-step sucrose gradient, containing 1 ml 55% (w/v) sucrose in 3 mM EDTA (pH 8.0) on the bottom of an ultracentrifuge tube and 5 ml 17% (w/v) sucrose on the top. The supernatant was subsequently centrifuged at 30,000 × *g *for 90 min to separate the membrane fraction from the cytosolic fraction. To membrane fractions equal volume of 3 mM EDTA (pH 8.0), and then 50% trichloroacetic acid (TCA) to the final concentration of 8% was added, and left overnight at 4°C. For protein precipitation, probes were centrifuged 60 min at 10,000 × *g *at 8°C, washed twice with acetone, each time spinning 15 min at 10,000 × *g*, air dried and final pellet was resuspended in 200 μl loading buffer. The protein concentration in the final preparations was determined using the Bradford kit (Bio-Rad). Secreted and membrane proteins of the Rt24.2 and the Rt2472 were separated by SDS-PAGE with 12% acrylamide and visualized by staining with Coomassie brilliant blue G-250.

### Protein sequencing

Membrane and extracellular protein fractions of Rt24.2 and Rt2472 separated by SDS-PAGE electrophoresis were transferred onto polyvinylidene difluoride (PVDF) membrane (Sequi-Blot; Bio-Rad) using the buffer containing 2.2% 3-(cyclohexylamino)-1-propanesulfonic acid (CAPS) (w/v), 10% methanol (v/v) (pH 11). Proteins were visualized by staining with Coomassie brilliant blue R-250, and interesting bands were excised from the membrane for the analysis. Protein sequencing was performed in BioCentrum sp. z o.o. Service lab in Cracow, Poland. Amino acids abstracted sequentially from the N-terminus in the form of phenylthiohydantoin derivatives (PTH) were analyzed using the automatic sequencer Procise 491 (Applied Biosystems, Foster City, CA, USA) and following standard manufacturer's protocols.

### Immunoblotting

Proteins separated by SDS-PAGE were transferred onto polyvinylidene difluoride (PVDF) membrane (Immobilon P; Millipore). Following transfer, the membrane was blocked with 3% (w/v) low fat milk in TBS buffer for 1 h, and incubated 1 h with rabbit polyclonal antibodies against PssB cytoplasmic protein [39] or PssN outer membrane protein [40] diluted 1:20000 and 1:40000, respectively. The membrane was washed 3 times for 10 min with TBS, and incubated for 2 h with 1:30000 dilution of alkaline phosphate-conjugated goat anti-rabbit IgG (Sigma). The membrane was visualized with alkaline phosphatase substrates (nitro tetrazolium blue and 5-bromo-4-chloro-3-indolylphosphate, NBT/BCIP, Roche) in a color development buffer.

### EPS and LPS isolation

For large-scale EPS isolation, 500-ml cultures of rhizobial strains were grown in 79CA medium with 1% glycerol for 5 days at 28°C in a rotary shaker. EPS was precipitated from supernatants with three volumes of cold ethanol. After centrifugation, the acidic EPS was dissolved and further fractionated by 2% hexadecyltrimethylammonium bromide (cetrimide) precipitation. The precipitate was dissolved in 1 M NaCl and reprecipitated with 3 volumes of ethanol. After the solubilization in water, the samples were dialyzed (12 kDa MWCO) against water, passed through the column with Dowex 50W × 8 [H^+^] to remove sodium ions and lyophilized. EPS samples were size-fractionated by column chromatography. Bio-Gel A-5m (Bio-Rad, Hercules, CA, USA) column (1.6 × 60 cm) equilibrated with sodium phosphate buffer (50 mM, pH 7.0) containing 100 mM sodium chloride as described in [71] was loaded with EPS samples. Fractions were collected and assayed for carbohydrates by the indole - sulphuric acid method. Total sugar content was calculated as glucose equivalents.

Prior to LPS isolation, bacterial cells were washed three times with 0.9% NaCl solution to remove extracellular polysaccharides. LPS was extracted using the hot phenol procedure and the aqueous phase was dialyzed against water. The water phase LPS was brought to 50 mM Tris-HCl, supplemented with 1 mM MgCl_2 _(pH 7.0), and treated with RNase A (500 units) for 6 h at 37°C, followed by proteinase K (0.1 mg/ml) digestion for 60 min at 60°C. The LPS preparations were pelleted by centrifugation at 105,000 × *g *for 4 h. To remove any attached glucans, LPS was purified by an extraction into 80% aqueous phenol and precipitation with 10 volumes of cold 95% ethanol. Finally, the precipitate was dissolved in carbonate buffer and submitted to polymyxin - agarose affinity column chromatography as described by Kannenberg and Carlson [72]. The LPS preparations eluted from polymyxin column by carbonate buffer containing 1% deoxycholate were used for GC-MS analysis, and were separated by 12.5% Tricine SDS-PAGE and visualized by silver staining [73].

### EPS and LPS analysis

The sugar composition of the degraded polysaccharides liberated from LPSs of the wild type and Rt2440 by mild acid hydrolysis (1% acetic acid, 100°C, 90 min) was determined by GC-MS analysis of their alditol acetates. For this, water soluble degraded polysaccharide obtained after lipid A centrifugation was subjected to reduction (NaBH_4_, 25°C, 90 min). For the determination of acid sugars, the samples were subjected to methanolysis at 85°C for 16 h in 1 M methanolic HCl, carboxyl reduction with NaBD_4_, hydrolysis with 2 M trifluoroacetic acid (TFA) for 4 h at 100°C, reduction with NaBD_4_, and acetylation. For the neutral and amino sugar analysis, the samples were hydrolyzed with 2 M TFA, N-acetylated prior to reduction with NaBD_4_, and acetylated.

The glycosyl composition analysis of EPS samples was performed after methanolysis, followed by trimethylsilylation as described in Vanderlinde et al. [74]. Part of the methanolysates was subjected to carboxyl reduction (NaBD_4_), hydrolysis in 2 N TFA, reduction and acetylation, as in the procedure described above for the acidic sugar determination in LPS. Monosaccharides in the form of alditol acetates and methyl glycosides of trimethylsilyl ethers were analysed by GC-MS on the Hewlett-Packard (5890) gas chromatograph interfaced to the 5971 mass selective detector using the 30 m HP-5MS capillary column (temperature program 150°C for 5 min, raised to 310°C at 5°C/min).

NMR spectroscopy - ^1^H experiments were recorded with the Varian Unity plus 500 instrument in D_2_O solutions at 70°C with acetone as an internal standard (d 2.225 ppm) using standard Varian software.

### Motility assay

*R. leguminosarum *motility assay was conducted in 0.3% M1 agar medium. 5 μl culture grown in liquid TY medium at 28°C for 24 h to an OD_600 _of 0.4 was stabbed into plates with M1 medium. To eliminate the flocculation of the *rosR *mutants, cell clumps were wiped and broken up on the inner surface of a glass tube using a sterile wooden stick. Then, the tube was left standing for 15 min so that the remaining clumps sunk to the bottom. The suspended cells from the top were taken carefully and, if needed, diluted down into TY to get the desired cell density (OD_600 _of 0.4). The plates were incubated at 28°C for 3 days, and bacterial growth from the point of inoculation was measured. Motility assay was done twice in triplicate.

### Biofilm formation assay - microtiter plate method

The biofilm formation assay was done according to method described by Rinaudi and Gonzalez [15]. Briefly, *R. leguminosarum *strains were grown in M1 medium supplemented with Dilworth's vitamins at 28°C for 48 h. The cultures were diluted to an OD_600 _of 0.4, inoculated into the polystyrene microplate wells in 100 μl aliquots, and incubated with agitation (100 rpm) at 28°C for 48 h. After this time, bacterial growth was assessed by measuring the OD_600_. The contents of wells were removed and each well was washed three times with 150 μl of 0.85% NaCl, stained for 15 min with 150 μl of 0.1% crystal violet, and then rinsed three times with water. Biofilm formation was quantified by the addition of 150 μl of 95% ethanol and measurement of the absorbance at 560 nm in a microplate reader. The experiment was performed in triplicate, repeated three times, and averaged.

### Confocal laser scanning microscopy

To visualize different stages of *R. leguminosarum *biofilm formation in a 4-day time-course experiment in polystyrene microplate wells, the inverted microscope Axiovert 200M equipped with LSM 5 Pascal head (with magnification 200x) was used. To obtain images of biofilm formation, bacterial cultures were stained with either Calcofluor (Sigma) or Bacterial Viability kit LIVE/DEAD *BacLight*™ (Invitrogen). Calcofluor was used for general visualization of the biofilm surface and structure, and two components of Bacterial Viability kit for the determination of a ratio of live (stained with Syto-9) to dead (stained with propidium iodide) cells in biofilm [75]. Bacterial cultures growing in TY medium for 48 h to an OD_600 _of 0.6 were diluted 1000-fold in M1 minimal medium supplemented with Dilworth's vitamins, and 100 μl of diluted cultures were added to each well and grown under static conditions at 28°C for up to 4 days. After 2 and 4 days, the contents of the wells were removed and each well was washed two times with 150 μl of sterile physiological saline solution, and then stained for 30 min with 100 μl of 25 μg/ml Calcofluor or 100 μl of 0.85% NaCl containing 5 μM Syto-9 and 30 μM propidium iodide. Next, dye solutions were removed and the wells were washed three times with 150 μl of 0.85% NaCl, covered by 30 μl of fresh portion of physiological saline solution, and observed in a microscope. This experiment was repeated two times. To analyze different parameters of biofilm, 36 images from 3 wells of individual strain were collected. The ratio of live to dead cells was calculated using the ImageJ 1.43e software (Wayne Rasband, NIH, USA). Images of biofilms stained with Syto-9 were analyzed to calculate several morphological parameters. The percentage of area covered by biofilm, a fractal dimension, and the length of coastline were calculated using ImageJ 1.43e software according to [76,77]. Three-dimensional images were reconstructed using the Laser Scanning Confocal Microscope LSC 5 PASCAL (Carl Zeiss, Germany) with 200x magnification.

### Plant tests

Red clover (*Trifolium pratense *cv. Diana) seeds were surface sterilized, germinated and grown on Fåhraeus medium [66] slants. 5-day-old seedlings were inoculated with bacterial suspensions at an OD_600 _of 0.2 (200 μl/plant), and grown under natural light supplemented with artificial light (14 h day at 24°C and 10 h night at 18°C) in a greenhouse. The clover plants were inspected for root nodule formation and harvested after 4 weeks. Wet and dry masses of clover shoots were estimated.

### Plant competition assay

For the competition assay, the Rt2472 and Rt2441 mutants, and the wild type Rt24.2 were collected from TY agar medium into sterile water to an OD_600 _of 0.1. The mutants and wild type suspensions were mixed in 1:1, 10:1, 100:1, and 1000:1 ratios, and 200 μl of each mixture were added per plant. Twenty seedlings were used for each treatment. 28 days after infection, nodules were surface sterilized, crushed in 20 μl of saline solution, and 10 μl portions were plated on 79CA agar plates supplemented with nalidixic acid or kanamycin, and incubated at 28°C for 3 days. Ninety nodules per each mixture were examined. Bacteria growing exclusively on the medium supplemented with nalidixic acid corresponded to the wild type strain, and those growing on the medium supplemented with kanamycin corresponded to the *rosR *mutants. The competitive ability of rhizobia was expressed as the percentage of the particular strain in the analyzed nodules.

### Assays for root attachment and growth on the root surface

Root attachment of the Rt2472 and the Rt24.2 carrying pHC60 with a constitutively expressed *gfp *[42] was assayed according to the method described by Williams et al. [18]. The strains were grown in 79CA to an OD_600 _of ~0.6, washed twice in sterile water, and resuspended in 25 mM phosphate buffer (pH 6.8) to a final OD_600 _of 0.06. 200 μl of a bacterial suspension was placed onto a slide with modified Fåhraeus medium containing a sterile germinated clover seedling with root ~2 cm long. The slides were incubated for 90 min at room temperature, and root attachment of tested strains was observed under confocal laser scanning microscopy. To study plant root invasion by the Rt2472 and the Rt24.2, clover seedlings ~2 cm long were placed on the top of microscope slides, which were previously covered with 2 ml Fåhraeus agar, and inoculated with 100 μl of bacterial suspension in sterile water of OD_600 _of 0.08 [42]. The slides with seedlings were placed in 50-ml culture tubes containing 5 ml of liquid Fåhraeus medium and covered loosely by sterile Whatman paper. To determine the efficiency of invasion, 25 plants inoculated with the particular strain were examined after 3, 4, 6, 8, and 10 days.

To determine quantitatively adhesion efficiency and the growth rate on clover roots by the Rt2472 and Rt24.2, the methods described by Fujishige et al. 2006 [78] were applied. For adhesion assay, three-day-old seedlings were inoculated by dipping their roots into bacterial suspensions of OD_600 _of 0.08 for 30 min or placed on Fåhraeus agar medium plates, inoculated by bacterial suspensions of OD_600 _of 0.08 (100 μl per seedling), and incubated for two days. The seedlings were placed on sterile Whatman paper to remove the excess of liquid, and subsequently were grown on Whatman paper wetted with liquid Fåhraeus medium for 48 h. Next, roots were washed overnight with sterile water containing 0.05% Tween-20 on a rocking platform shaker to remove loosely associated cells. After removing the excess of liquid, the roots were weighed. To determine the number of attached bacteria, the root of each seedling was homogenized in 300 μl of water and root homogenate was plated in dilutions on 79CA plates for colony counting.

## Authors' contributions

MJ performed genetic analyses of the *rosR *mutants, carried out experiments concerning their phenotype characterization and plant experiments, and drafted the manuscript. JK conducted EPS and LPS analyses, TP performed microscope images and parameter analyses of biofilm. AS discussed the results and elaborated the final version of manuscript. All authors read and approved the final version of the manuscript.

## Supplementary Material

Additional file 1 **Figure S1 - Western blotting analysis of membrane and extracellular protein fractions of the *R. leguminosarum *wild type and the *rosR *mutant (Rt2472) with polyclonal antisera against PssB (A) and PssN (B)**. The migration positions of molecular mass markers are shown. Lines 1-6: extracellular protein fractions isolated from 10 ml of: Rt24.2 TY culture supernatant (1), Rt2472 TY culture (2), Rt24.2 M1 culture (3), Rt24.2 M1 culture with 5 μM exudates (4), Rt2472 M1 culture (5), Rt2472 M1 culture with 5 μM exudates (6). Lines 7-12: 6 μg of membrane protein fractions isolated from: Rt24.2 cells grown in TY (7), Rt2472 cells grown in TY (8), Rt24.2 cells grown in M1 (9), Rt24.2 cells grown in M1 with 5 μM exudates (10), Rt2472 cells grown in M1 (11), Rt2472 cells grown in M1 with 5 μM exudates (12), Lines: 13 and 14 - cytoplasmic protein fractions of Rt24.2 and Rt2472, respectively, grown in M1 medium. Click here for file
