# Supplementary figures and images for: Rhizobium leguminosarum bv. trifolii rosR is required for interaction with clover, biofilm formation and adaptation to the environment
Source: BMC Microbiol. 2010 Nov 11;10:284. doi: 10.1186/1471-2180-10-284 (PMC2996380; doi:10.1186/1471-2180-10-284)

**A**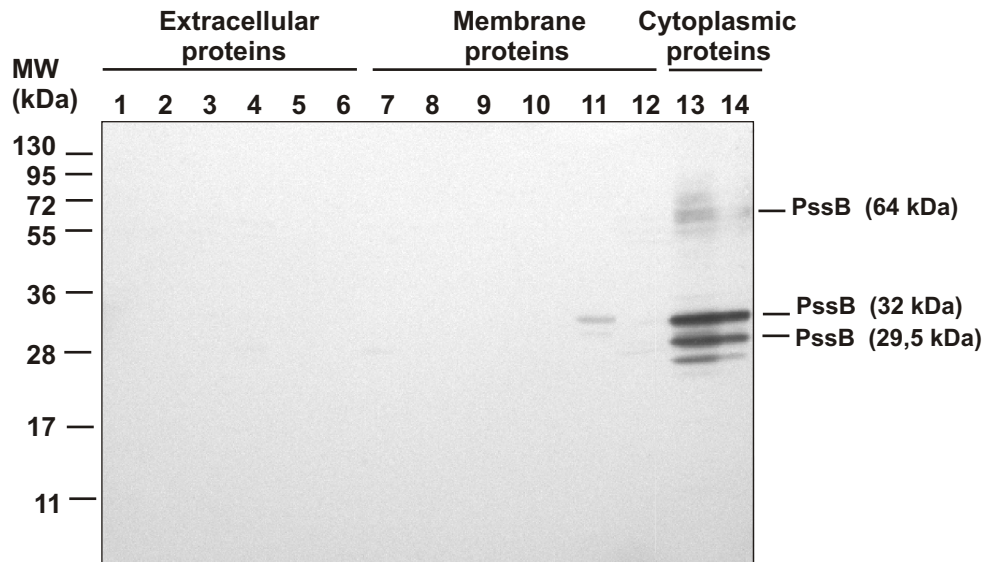**B**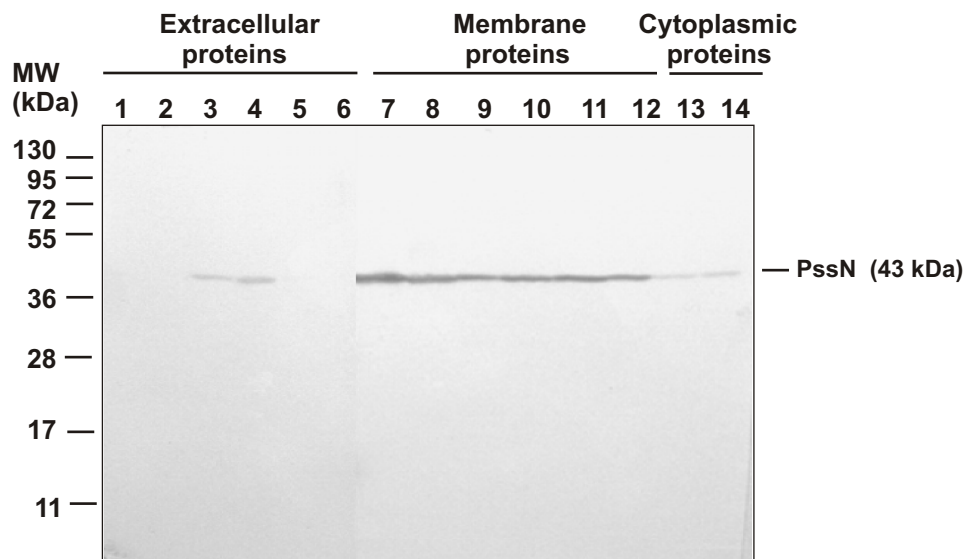

Supplement: Additional file 1 — Figure S1 - Western blotting analysis of membrane and extracellular protein fractions of the R. leguminosarum wild type and the rosR mutant (Rt2472) with polyclonal antisera against PssB (A) and PssN (B). The migration positions of molecular mass markers are shown. Lines 1-6: extracellular protein fractions isolated from 10 ml of: Rt24.2 TY culture supernatant (1), Rt2472 TY culture (2), Rt24.2 M1 culture (3), Rt24.2 M1 culture with 5 μM exudates (4), Rt2472 M1 culture (5), Rt2472 M1 culture with 5 μM exudates (6). Lines 7-12: 6 μg of membrane protein fractions isolated from: Rt24.2 cells grown in TY (7), Rt2472 cells grown in TY (8), Rt24.2 cells grown in M1 (9), Rt24.2 cells grown in M1 with 5 μM exudates (10), Rt2472 cells grown in M1 (11), Rt2472 cells grown in M1 with 5 μM exudates (12), Lines: 13 and 14 - cytoplasmic protein fractions of Rt24.2 and Rt2472, respectively, grown in M1 medium. [file 1471-2180-10-284-S1.PDF]
